# Supplementary material for: Role of monkeys in the sylvatic cycle of chikungunya virus in Senegal
Source: Nat Commun. 2018 Mar 13;9:1046. doi: 10.1038/s41467-018-03332-7 (PMC5849707; doi:10.1038/s41467-018-03332-7)
Supplement: Supplementary file 1 — Supplementary Information [file 41467_2018_3332_MOESM1_ESM.pdf]

Supplementary Information for Role of monkeys in the sylvatic cycle of chikungunya virus in Senegal

Benjamin M. Althouse, Mathilde Guerbois, Derek A. T. Cummings, Ousmane M. Diop, Ousmane Faye, Abdourahmane Faye, Diawo Diallo, Bakary Djilocalisse Sadio, Abdourahmane Sow, Oumar Faye, Amadou A. Sall, Mawlouth Diallo, Brenda Benefit, Evan Simons, Douglas M. Watts, Scott C. Weaver, and Kathryn A. Hanley

Supplementary Figures

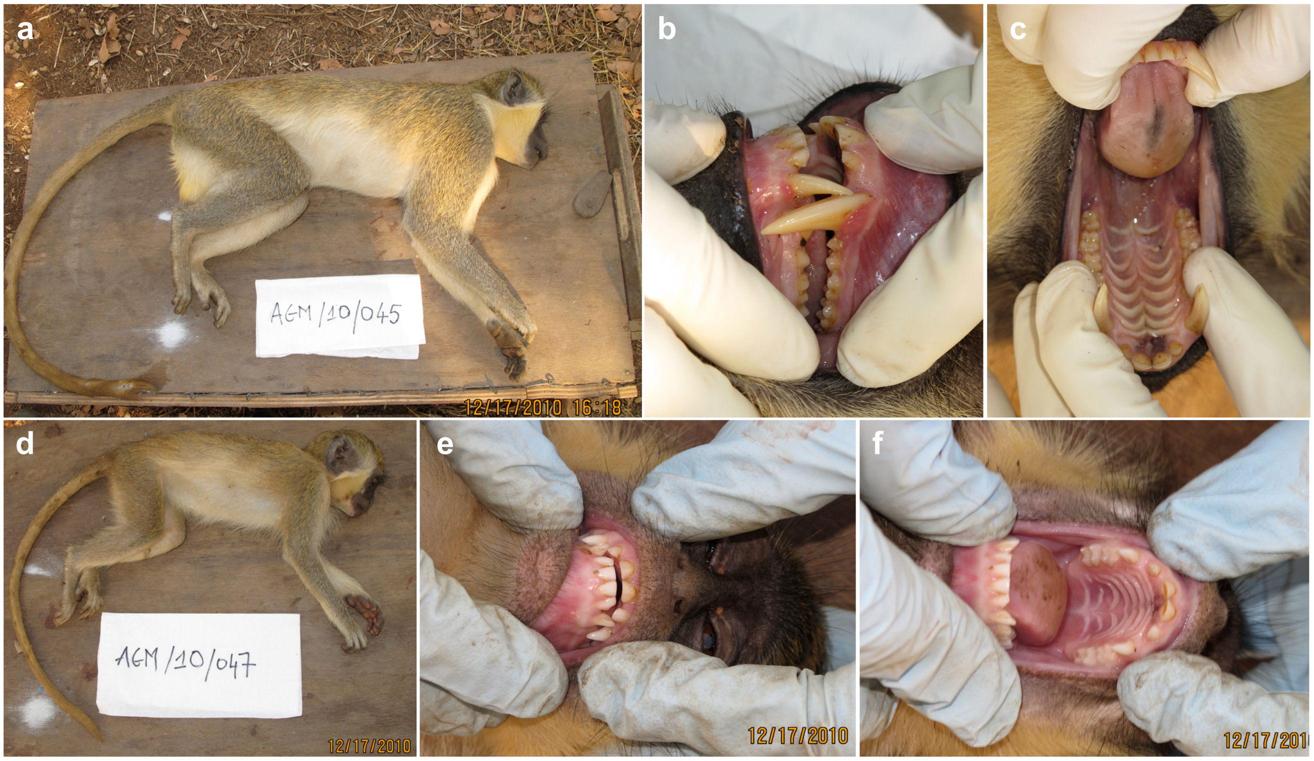

Supplementary Figure 1: **Example *Chlorocebus sabaeus*** Figure shows an example 5 year old male (panels **a-c**) and 3 month old male (panels **d-f**) *C. sabaeus*.

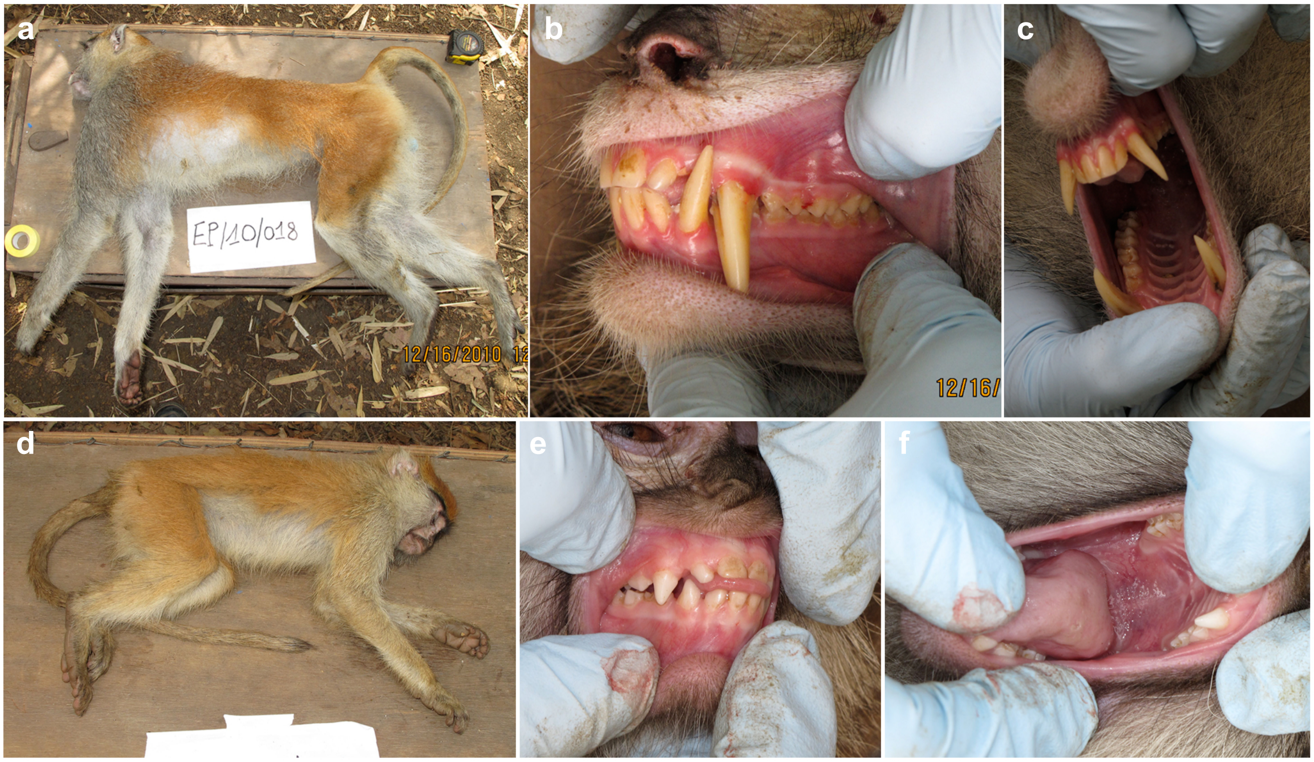

Supplementary Figure 2: **Example *Erythrocebus patas*** Figure shows an example 4 year old male (panels **a-c**) and 7 month old male (panels **d-f**) *E. patas*.

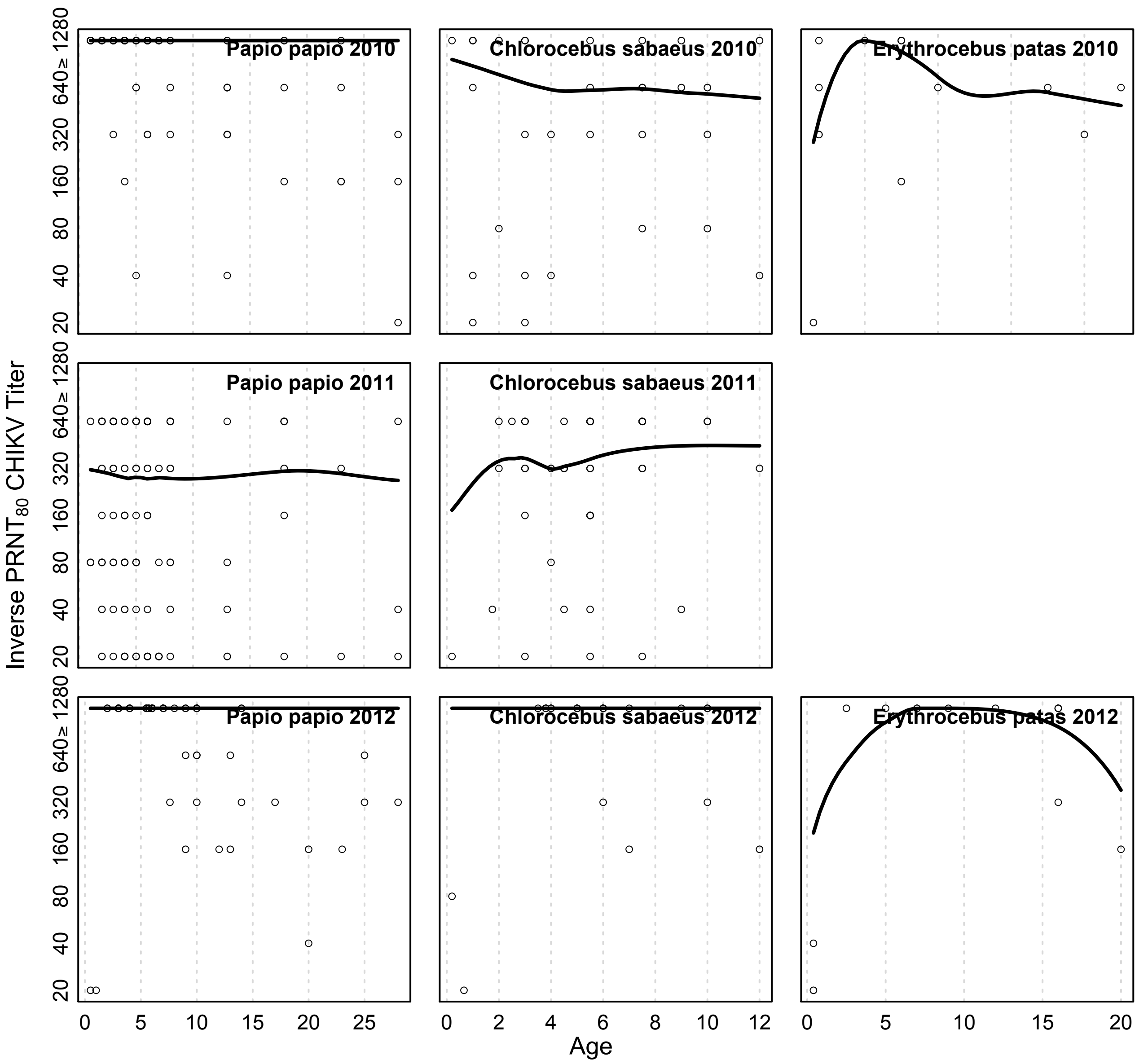

Supplementary Figure 3: **Inverse $PRNT_{80}$ titer by age and species**.

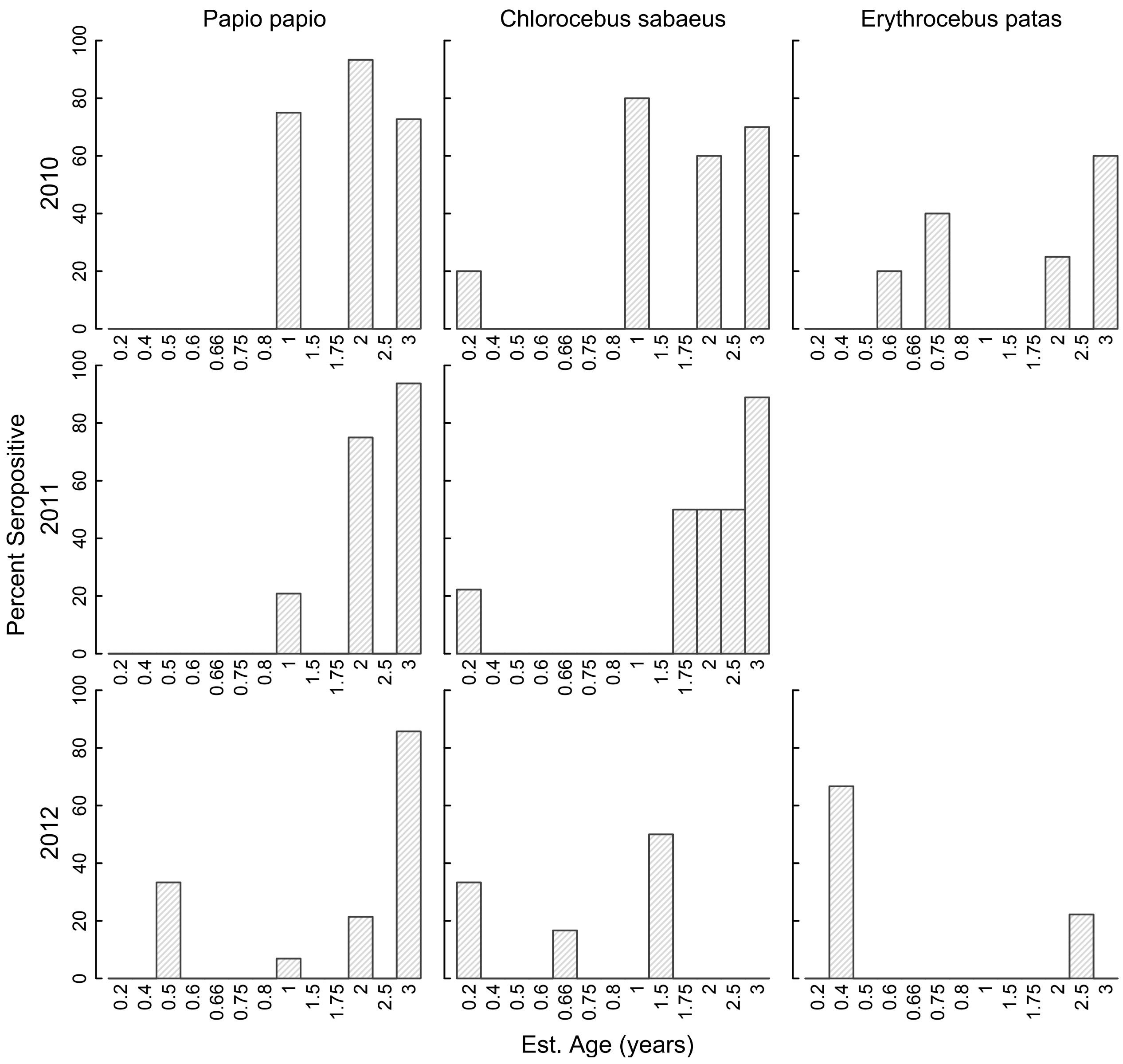

Supplementary Figure 4: **Seropositivity in Young Monkeys** Figure shows seropositivity for monkeys under 3 years of age.

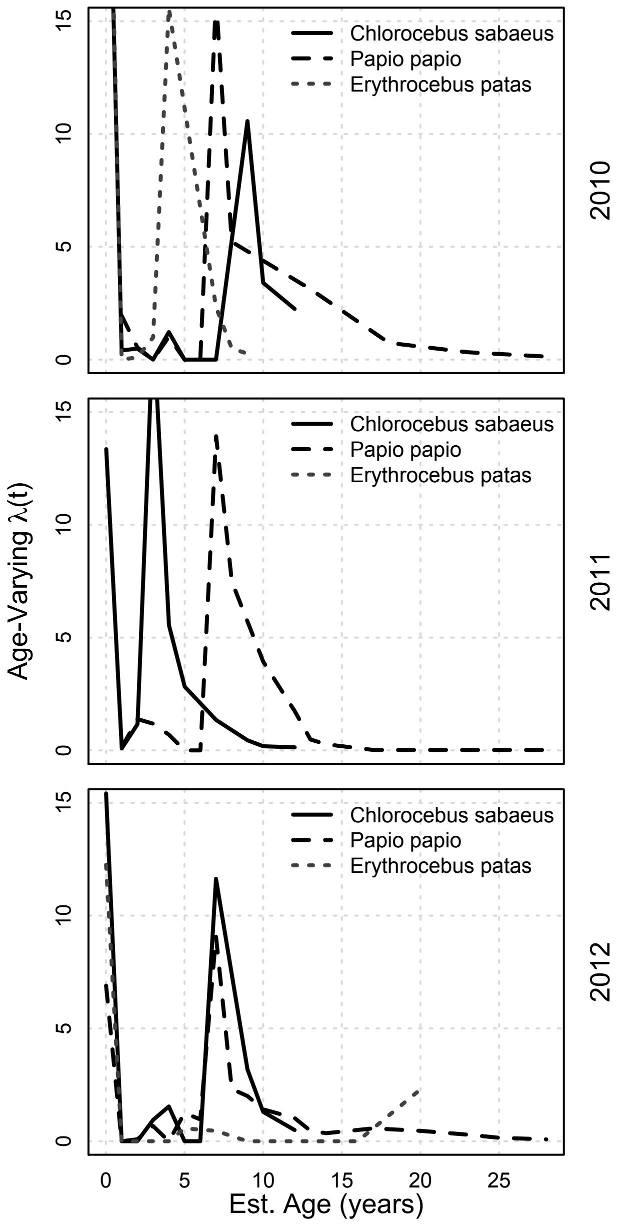

Supplementary Figure 5: **Age-Varying Force of Infection** Figure shows estimates of $\lambda(t)$ by age category.

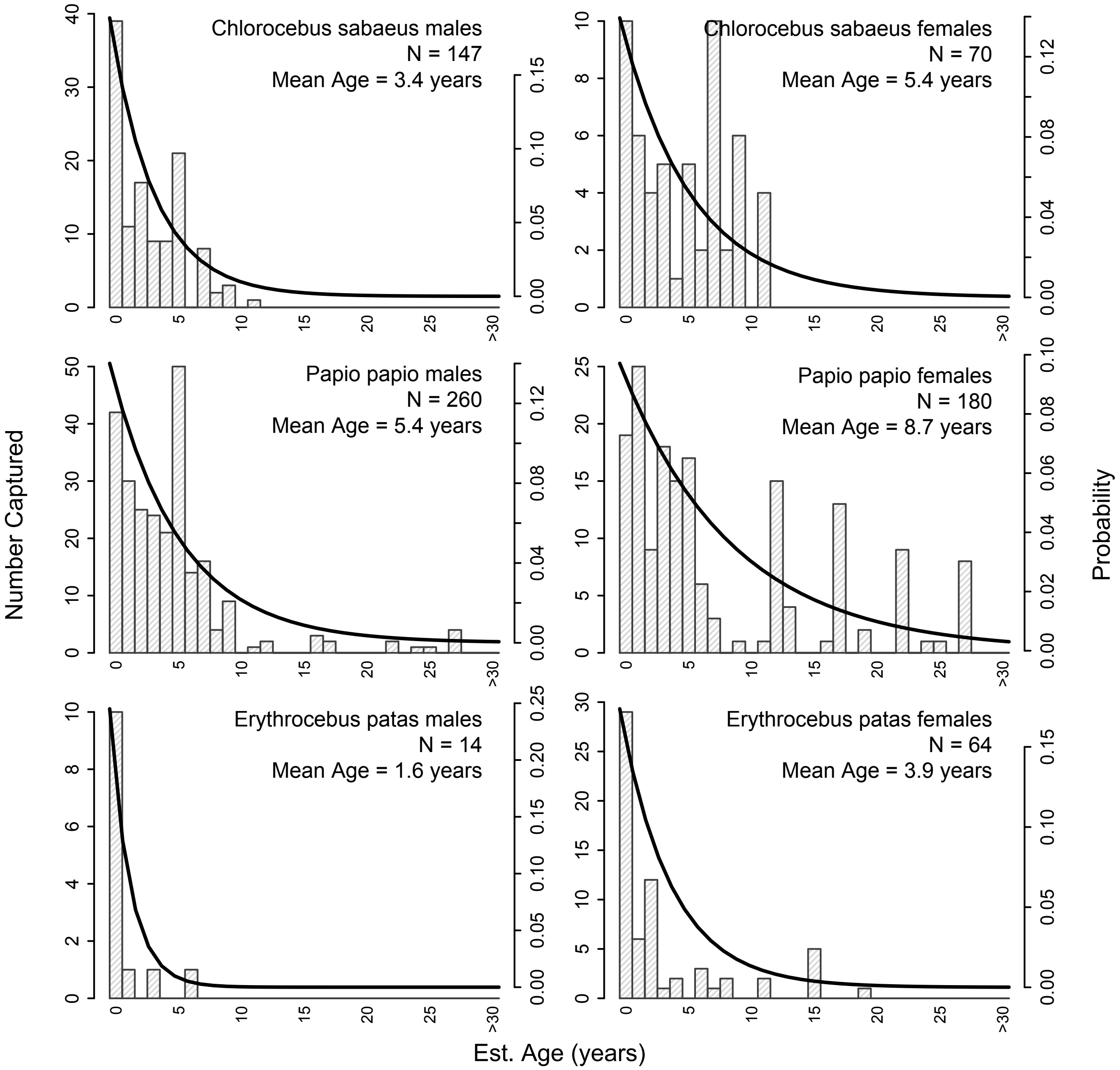

Supplementary Figure 6: **Age-Distributions by Sex** Supplementary Figure shows the age distributions of collected monkeys by sex. An exponential distribution with rate equal to observed mean age is overlaid.

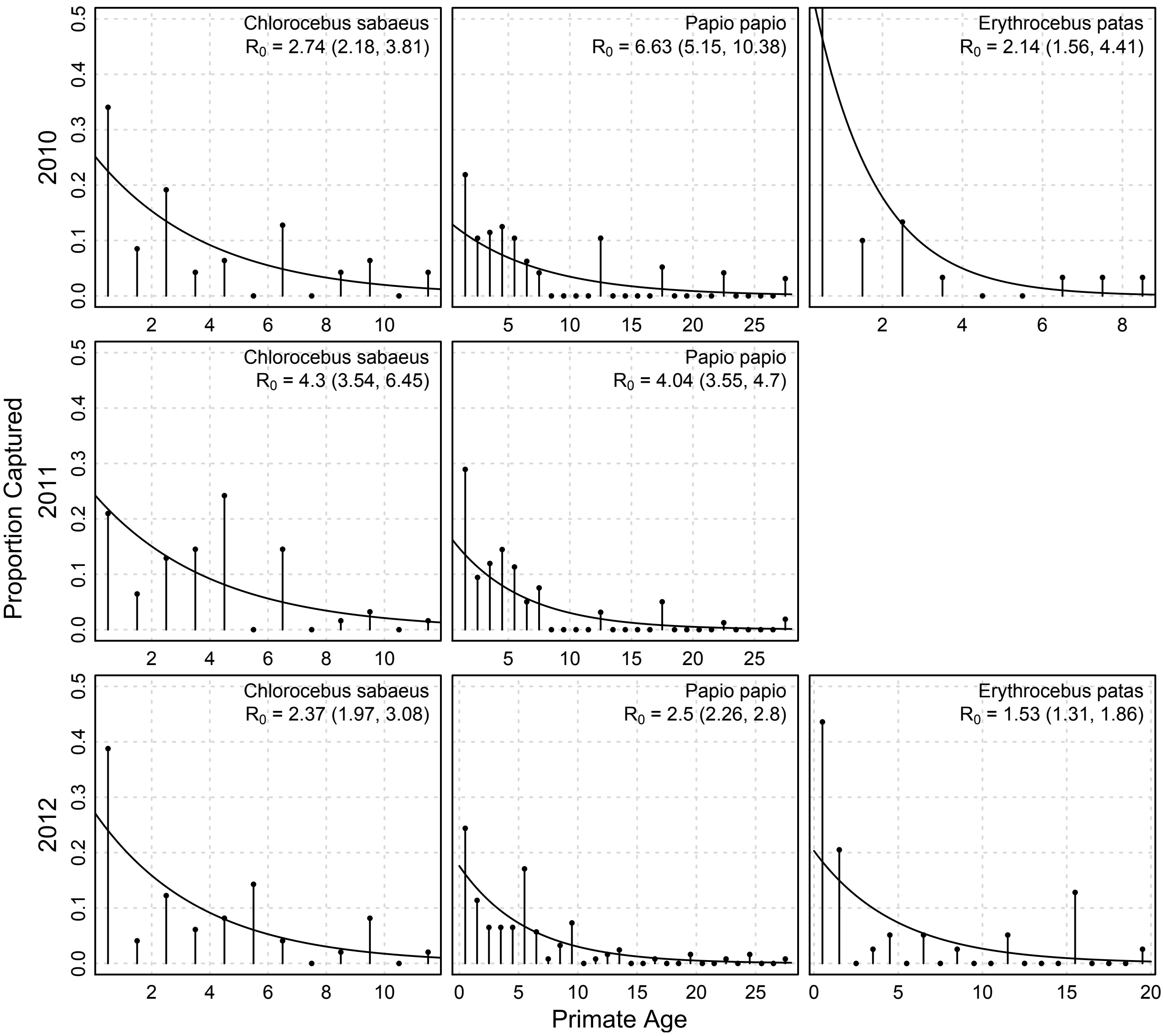

Supplementary Figure 7: **Age-Distributions and Basic Reproductive Number** Figure shows the age distributions of collected monkeys by year. An exponential distribution with rate equal to observed mean age used to calculate R_0 is overlayed.

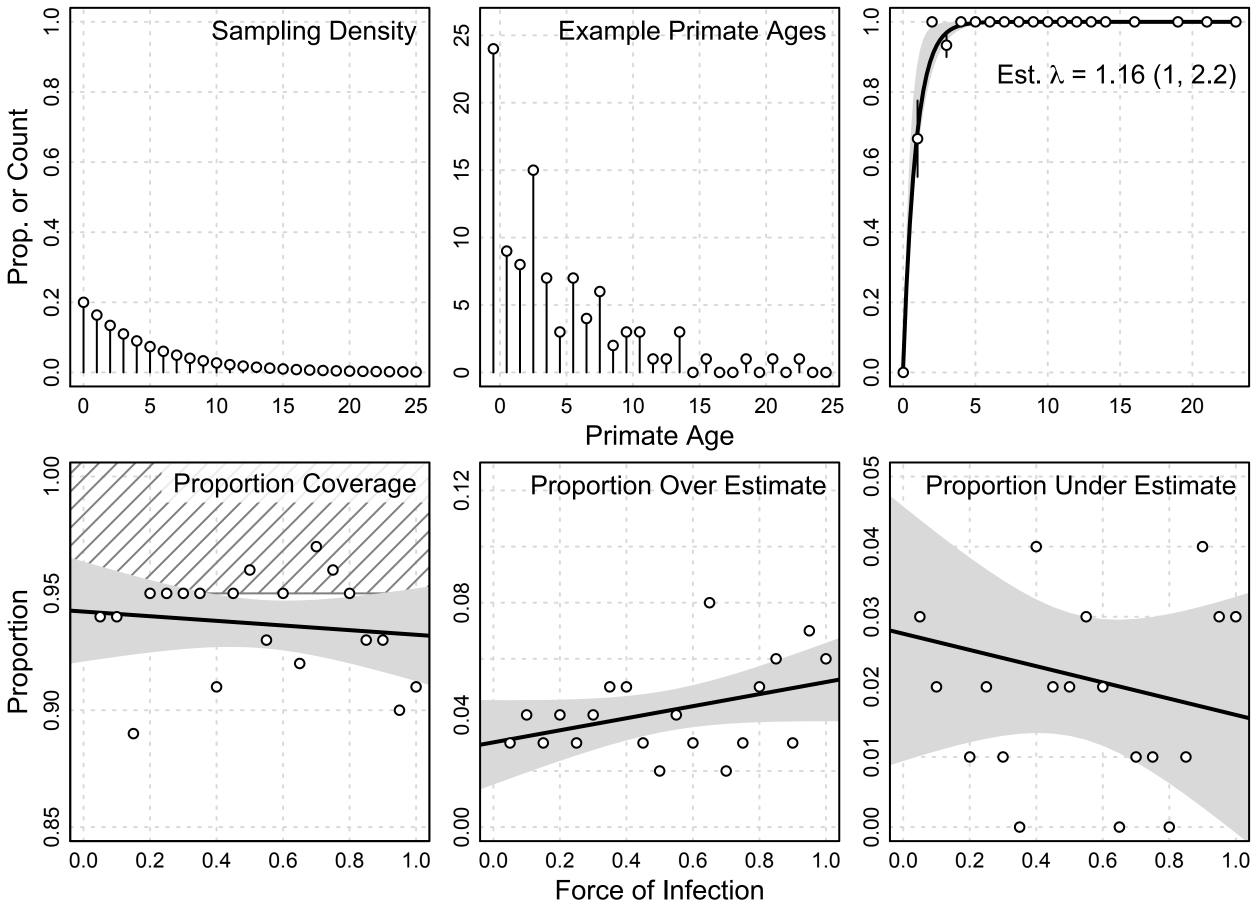

Supplementary Figure 8: **Summary of Sensitivity Analyses: Exponential Distribution** Figure summarizes the sensitivity analysis, assuming an exponential age bias in collections (rate 1/5 years). The top row shows the sampling distribution, and example primate sample and estimation of force of infection (left to right). The bottom row shows the proportion of estimated confidence intervals for $\lambda(t)$ that contain the true value, the proportion of estimated $\lambda(t)$ that were greater than the truth and smaller than the truth (left to right). Dark lines are linear regressions and grey bands are the uncertainty in the slope.

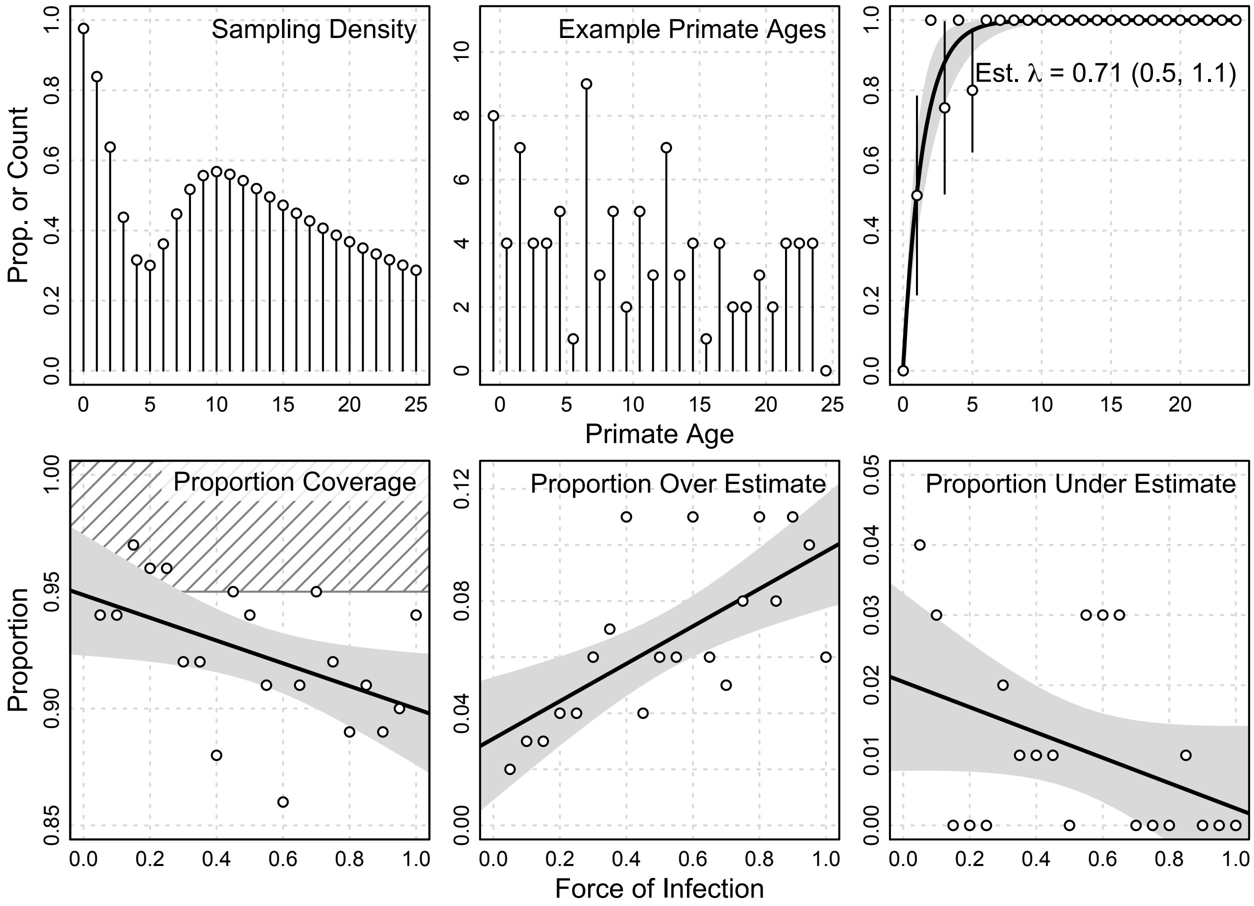

Supplementary Figure 9: **Summary of Sensitivity Analyses: Alternative Distribution** Figure panels are identical to Supplementary Figure 8, but with an alternative sampling distribution.

Supplementary Tables

	C. sabaeus	*E. patas*	Papio papio
Bafoundou	0	1	55
Bambakolong	0	16	0
Bambara tickendala	39	0	100
Bamboto	0	7	0
Diaguiri	61	10	10
Douta	7	0	163
Kenyoto	8	0	0
Komewoto	49	0	0
Ngary	0	0	48
Samecouta	11	30	0
Sekoto	12	0	0
Silly	13	0	0
Silly (PC1)	19	10	0
Sougnouguiri	0	4	0
Walydionkounda	0	0	64

Supplementary Table 1: **Monkeys Collected by site** Table shows the numbers of monkeys collected in each site across all years.

Age Class	N	I1	I2	C	P3	P4	M1	M2	M3	Sum Wear	E Age Gilgil	E Age Mikumi	E+W Age
F1a	3	D/D	D/D	D/D	D/D	A/D	N/N	N/N	N/N	0	0.8-1.6	<3.4	1
F1b	16	D/D	D/D	D/D	D/D	D/D	N/N	N/N	N/N	0	0.9-1.6	<3.4	1
F2a	5	D/D	D/D	D/D	D/D	D/D	N-A/E-Y1-2	N/N	N/N	0-2	1.6-2.8	<3.4	2
F2b	20	D/D	D/D	D/D	D/D	D/D	Y1/Y1	N/N	N/N	2	1.7-2.8	<3.4	2
F2c	1	D/D	D/D	D/D	D/D	D/D	Y3/Y3	N/N	N/N	6	1.7-2.8	<3.4	2
F3a	5	E-P/D	D-M/D-M	D/D	D/D	D/D	Y1/Y1-3	N/N	N/N	2-4	2.8-3.3	3.4-4.1	3
F3b	5	P/P	D-M/D	D-M/D	D/D	D/D	Y1-2/Y1-2	N/N	N/N	2-4	2.8-3.3	3.4-4.1	3
F4a	2	P/P	D/E-A	D/D	D/D	D/D	A-Y2/A-Y1	N/N	N/N	0-3	3.25	4.2	3.5
F4b	5	P/P	M-A/P	D/D	D/D	D/D	Y1-2/Y2-3	N/N	N/N	4-5	3.25	4.2-4.3	3.5
F4c	3	P/P	P/P	D/D	D/D	D/D	Y1-2/Y1-2	N/N	N/N	4-5	3.25-3.7	4.2	3.7
F4d	1	P/P	P/P	D/E	D/D	D/D	Y2/Y2	N/N	N/N	4	3.25-3.7	4.2	3.7
F5a	2	P/P	P/P	D/E	D/D	D/D	Y2/Y2-3	N/E	N/N	4-5	3.7-3.8	4.2-4.6	4
F5b	1	P/P	P/P	D/E	D/D	D/D	Y2/Y2	E/E	N/N	4	3.7-3.8	4.2-4.6	4
F5c	1	P/P	P/P	D/E	A/A	D/M	Y2/Y2	N/E	N/N	4	5.3	5	5.3
F5d	2	P/P	P/P	D/E	A/E	A/A	Y2-3/Y3	A-Y1/A	N/N	5-6	5.5	4.2-4.6	5.5
F5e	1	P/P	P/P	D/D	P/D	P/D	Y2/Y3	Y1/Y1	N/N	7	5.3	4.2-4.6	5.5
F5F	1	P/P	P/P	D/E	D/E	D/D	Y3-4/Y3-4	Y1/Y1	-/-	8	5.3	4.2-4.6	5.5
F6a	3	P/P	P/P	E/E	D/E-D	D/D	Y2-4/Y2-4	E-A/E-Y1	N/N	4-8	5.3	4.2-4.6	6
F6b	3	P/P	P/P	E/E	A-P/A-P	E-A/A-P	Y2-4/Y3-4	A-Y1/A-Y1	N/N-E	5-10	5.3	4.2-4.6	6
F6c	4	P/P	P/P	E-A/E-P	A/A-P	A-P/A-P	Y2-4/Y3-4	A-Y1/Y1-2	N/N	6-10	5.3	4.2-4.6	6
F6d	1	P/P	P/P	P/P	P/A	P/A	Y2-3/Y3	Y1/Y1	N/N	6	5.3	4.2-4.6	6
F6e	20	P/P	P/P	P/P	P/P	P/P	Y3-5/Y3-5	Y1-3/Y1-3	N/N	8-16	5.3	4.2-4.6	7
F7a	2	P/P	P/P	P/P	P/P	P/P	Y3-4/Y4-5	Y1-2/Y1-3	N/E	9-14	7	6.4-8.1	7.6
F7b	5	P/P	P/P	P/P	P/P	P/P	Y4-10/Y4-10	Y2-5/Y3-5	E-A/E-Y1	14-30	7	6.4-8.1	7.6
F8a	4	P/P	P/P	P/P	P/P	P/P	Y4-5/Y4-5	Y3-4/Y3-4	Y1/Y1	17-20+	>7	>8.1	8
F8b	8	P/P	P/P	P/P	P/P	P/P	Y5-6/Y5-6	Y3-5/Y2-5	Y1-2/Y1-3	19-23	>7	>8.1	10
F9a	4	P/P	P/P	P/P	P/P	P/P	Y5/Y6-7	Y4-5/Y4-5	Y1-3/Y3	24-26	>7	>8.1	12
F9b	5	P/P	P/P	P/P	P/P	P//P	Y7-8/Y5-7	Y4-5/Y3-5	Y1-2/Y2	24	>7	>8.1	13
F10	9	P/P	P/P	P/P	P/P	P/P	Y6-8/Y6-8	Y4-5/Y4-6	Y1-3/Y2-3	25-30	>7	>8.1	14
F11	6	P/P	P/P	P/P	P/P	P/P	Y6-7/Y7-8	Y4-7/Y5-7	Y3-4/Y4	31-34	>7	>8.1	17
F12	4	P/P	P/P	P/P	P/P	P/P	Y7-9/Y7-9	Y5-8/Y7-8	Y2-3/Y2-4	34-37	>7	>8.1	20
F13	9	P/P	P/P	P/P	P/P	P/P	Y7-9/Y8-10	Y6-9/Y5-9	Y4-6/Y4-7	37-48	>7	>8.1	23
F14	4	P/P	P/P	P/P	P/P	P/P	Y10-11/Y-11	Y7-8/Y7-10	Y3-6/Y9	37-48+	>7	>8.1	25
F15	1	P/P	P/P	P/P	P/P	P/P	Y10/Y10	Y10/Y10	Y8/Y8	54	>7	>8.1	26
F16	6	P/P	P/P	P/P	P/P	P/P	Y11/Y11	Y10-11/Y9-11	Y9-11/Y8-11	56-66	>7	>8.1	28

Supplementary Table 2: **Estimated age classes of *P. papio* Females** Monkeys were sorted into age classes based on the tooth eruption and degree to which dentine is exposed on molar occlusal surfaces. E Age refers to age based on dental eruption data and is given in months. E+W age is based on both dental eruption and molar wear and is given in years. Abbreviations given for teeth are: D=deciduous fully erupted, P=permanent fully erupted, E=gingival emergence, M=mesial half erupted, A=almost fully erupted, N=not erupted, and Y=molar present. Eruption and wear stages for upper teeth are given above and for lower teeth below the slanted line.

Age Class	N	I1	I2	C	P3	P4	M1	M2	M3	Sum Wear	E Age Gilgil	E Age Mikumi	E+W Age
M1a	1	D/D	D/D	D/D	D/D	N/N	N/N	N/N	N/N	0	0.5-0.75	<3.4	0.5
M1b	5	D/D	D/D	D/D	D/D	dA/dA-D	N/N	N/N	N/N	0	0.75-1.7	<3.4	1
M1c	38	D/D	D/D	D/D	D/D	D/D	N/N	N/N	N/N	0	0.8-1.7	<2.1	1
M2a	1	D/D	D/D	D/D	D/D	D/D	N/E	N/N	N/N	0	0.8-1.7	2.1	2
M2b	8	D/D	D/D	D/D	D/D	D/D	E-A/A-Y1	N/N	N/N	0-2	0.8-1.7	2.1	2
M2c	25	D/D	D/D	D/D	D/D	D/D	A-Y1-2/Y1-2	N/N	N/N	1-4	1.7-2.7	2.1-3.5	2.5
M3a	2	E/D	D/D	D/D	D/D	D/D	A-Y1/Y1	N/N	N/N	2	2.7	3.7	3
M3b	1	D/E	D/E	D/D	D/D	D/D	Y1/Y1	N/N	N/N	2	2.7	3.4	3
M3c	5	P/P	D-M/D-M	D/D	D/D	D/D	Y1/Y1	N/N	N/N	1-2	2.7	3.7	3
M4a	7	P/P	D-E/E-A	D/D	D/D	D/D	Y1-3/Y1-3	N/N	N/N	2-6	3.3	3.5-4.3	3.5
M4b	11	P/P	D/P	D/D	D/D	D/D	Y1-2/Y1-3	N/N	N/N	2-5	3.3-5.1	3.5-4.3	3.5
M4c	9	P/P	M-A/P	D/D	D/D	D/D	Y1-3/Y1-3	N/N	N/N	2-5	3.3	4.3	4
M4d	10	P/P	P/P	D/D	D/D	D/D	Y1-3/Y1-3	N/N	N/N	2-7	3.3	4.3	4
M5a	1	P/P	D/P	D/D	P/D	D/D	Y3/Y-	N/E	N/N	3+	5.2	4.3-4.7	5
M5b	6	P/P	P/P	D/D	D/D	D/D	Y1-4/Y1-4	N-E/N-A	N/N	2-8	5.2	4.3-4.7	5
M5c	6	P/P	P/P	D/D	E/D	D/D	Y1-3/Y2-4	N-Y/E	N/N	4-8	5.2	4.3-4.7	5.5
M5d	3	P/P	P/P	D/D	P/D	D/D	Y3/Y3-4	E-Y1/Y1	N/N	6-8	5.2	4.3-4.7	5.5
M5e	4	P/P	P/P	D/D-E	A-P/D-P	D-A/D-P	Y3-4/Y3-4	E-Y1/E-Y1	N/N	6-9	5.2-6	4.75-5.7	5.7
M5f	9	P/P	P/P	D/E	P/D-P	P/D-P	Y3-5/Y3-5	Y1/Y1-2	N/N	6-13	5.2-6	4.75-5.7	5.8
M6a	7	P/P	P/P	M-E/E-P	A-P/E-P	A-P/A-P	Y2-5/Y2-4	Y1/Y1-3	N/N	6-13	6	6.3	6
M6b	4	P/P	D-E/P	P/A-P	P/P	P/P	Y1-3/Y1-4	Y1-3/Y1-2	N/N	2-9	6	6.3	6
M6c	10	P/P	P/P	D-E/E-P	E-P/E-P	P/E-P	Y3-4/Y3-4	Y1-2/Y1-2	N/N	8-12	6	6.3	6
M6d	5	P/P	A-P/P	A-P/A-P	A-P/A-P	A-P/A-P	Y3-4/Y3-4	Y1-2/Y1-3	N/N	8-13	6	6.3	6
M6e	16	P/P	P/P	P/P	P/P	P/P	Y2-5/Y3-5	Y1-3/Y1-3	N/N	8-14	6	6.3	6
M7a	4	P/P	P/P	P/P	A-P/P/P	A-P/P	Y1-3/Y3-5	Y1-3/Y1-3	N-Y1-3/E	6-14	7.8	6.4	7
M7b	9	P/P	P/P	P/P	P/P	P/P	Y3-7/Y4-7	Y1-4/Y1-3	N/E	10-18	7.8	6.4	7
M8a	5	P/P	P/P	A-P/P	A-P/P	A-P/P	Y2-5/Y3-5	Y1-3/Y1-4	E-A/E-Y1	9-16	>7.8	>8.2	9
M8b	17	P/P	P/P	P/P	P/P	P/P	Y3-5/Y4-5	Y2-3/Y2-4	Y1-2/Y1-2	14-20	>7.8	>8.2	10
M9	7	P/P	P/P	P/P	P/P	P/P	Y5-7/Y5-7	Y3-4/Y3-4	Y1-3/Y1-3	21-24	>7.8	>8.2	12
M10	4	P/P	P/P	P/P	P/P	P/P	Y7-8/Y6-8	Y-5/Y3-5	Y3/Y1-3	27	>7.8	>8.2	14
M11	4	P/P	P/P	P/P	P/P	P/P	Y5-8/Y7-8	Y4-7/Y4-7	Y2-4/Y2-4	29-33	>7.8	>8.2	17
M13	3	P/P	P/P	P/P	P/P	P/P	Y7-8/Y9-10	Y5-8/Y6-8	Y4-5/Y2-5	38-39	>7.8	>8.2	23
M14	1	P/P	P/P	P/P	P/P	P/P	Y8/Y8	Y7/Y8	Y5/Y6-7	42-43	>7.8	>8.2	25
M15	1	P/P	P/P	P/P	P/P	P/P	Y9/Y11	Y8/Y10	Y7-8/Y10	54	>7.8	>8.2	26
M16	5	P/P	P/P	P/P	P/P	P/P	Y10-11/Y10-11	Y10-11/Y10-11	Y10/Y9-11	60-65	>7.8	>8.2	28

Supplementary Table 3: **Estimated age classes of *P. papio* Males** Monkeys were sorted into age classes based on the tooth eruption and degree to which dentine is exposed on molar occlusal surfaces. E Age refers to age based on dental eruption data and is given in months. E+W age is based on both dental eruption and molar wear and is given in years. Abbreviations given for teeth are: D=deciduous fully erupted, P=permanent fully erupted, E=gingival emergence, M=mesial half erupted, A=almost fully erupted, N=not erupted, and Y=molar present. Eruption and wear stages for upper teeth are given above and for lower teeth below the slanted line.

Age Class	N	I1	I2	C	P3	P4	M1	M2	M3	Sum Wear	E Age Turner	E Age Bolter	E+W Age
F1	2	D/D	D/D	D/D	D/D	D/D	N/N	N/N	N/N	0	0-2.5	0-3	0.25
F2a	1	D/D	D/D	D/D	D/D	D/D	N/Y	N/N	N/N	0	8-12	12-14	1
F2b	7	D/D	D/D	D/D	D/D	D/D	E-Y1/E-Y1	N/N	N/N	1-2	8-12	12-14	1
F3	2	E/D	A-Y3	D/D	D/D	D/D	Y1/N-Y1	N/N	N/N	1-2	12-15	22-27	1.5
F4	1	P/P	D/D	D/D	D/D	D/D	Y1/Y1	N/N	N/N	2	15-18	22-27	1.5
F5	1	P/P	P/P	M-D/M-D	D/D	D/D	E-Y1/A-Y1-3	N/A-Y1	N/N	2-8	20-24	26-31	2
F6	4	P/P	P/P	P/P	P/P	P/P	Y1-4/Y1-4	Y1-4/Y1-4	N/N	4-16	30-36	32-40	3
F7	3	P/P	P/P	P/P	P/P	P/P	Y3-4/Y3-4	Y1/Y1-2	E/E-Y1	8-11	36-40	38-41	3.5
F8	7	P/P	P/P	P/P	P/P	P/P	Y4-5/Y4-7	Y2-4/Y2-3	A-Y1/A-Y1	16-18	>48	>38	4
F9	4	P/P	P/P	P/P	P/P	P/P	Y5-6/Y5	Y3-4/Y4-5	Y1-3/Y1-4	20-26	>48	>38	5
F10	7	P/P	P/P	P/P	P/P	P/P	Y4-8/Y7-8	Y2-7/Y5-6	Y2-4/Y3-4	26-33	>48	>38	6
F11a	2	P/P	P/P	P/P	P/P	P/P	Y7-8/Y9-10	Y6-7/Y9-10	Y3-7/Y8	42-49	>48	>38	7
F11b	2	P/P	P/P	P/P	P/P	P/P	Y9/Y9	Y7-8/Y7-8	Y5/Y5	44	>48	>38	8
F12	6	P/P	P/P	P/P	P/P	P/P	Y10/Y7-10	Y5-9/Y6-9	Y3-7/Y5-7	37-51	>48	>38	10
F13	4	P/P	P/P	P/P	P/P	P/P	Y11-12/Y10	Y10-11/Y11	Y7/Y7	48-50	>48	>38	12

Supplementary Table 4: **Estimated age classes of *C. sabaeus* Females** Monkeys were sorted into age classes based on the tooth eruption and degree to which dentine is exposed on molar occlusal surfaces. E Age refers to age based on dental eruption data and is given in months. E+W age is based on both dental eruption and molar wear and is given in years. Abbreviations given for teeth are: D=deciduous fully erupted, P=permanent fully erupted, E=gingival emergence, M=mesial half erupted, A=almost fully erupted, N=not erupted, and Y=molar present. Eruption and wear stages for upper teeth are given above and for lower teeth below the slanted line.

Age Class	N	I1	I2	C	P3	P4	M1	M2	M3	Sum Wear	E Age	E Age	E+W Age
M1	17	D/D	E-D/D	D/D	D/D	D/D	N/N	N/N	N/N	0	0.25	0.33	0.2
M2a	6	D/D	D/D	D/D	D/D	D/D	N-E/N	N/N	N/N	0	0.6-1	0.6-1	0.66
M2b	4	D/D	D/D	D/D	D/D	D/D	E-A/E-A	N/N	N/N	0	0.6-1	0.6-1	0.8
M2c	13	D/D	D/D	D/D	D/D	D/D	Y1/Y1	N/N	N/N	1-2	0.6-1	0.6-1	1
M2d	1	D/D	D/D	D/D	D/D	D/D	Y2/Y2	Y1/Y1	N/N	6	0.6-1	0.6-1	1
M3	4	A-P/D-P	M-D/M-D	D/D	D/D	D/D	Y1/Y1	N/N	N/N	2	1-1.5	1.8-2.2	1.5
M4	4	P/P	E-P/E-P	D/D	D/D	D/D	A-Y1/Y1-2	N/N	N/N	2-3	1.6-2	1.8-2.2	2
M5	3	P/P	E-P/P	D/D	D/D	D/D	Y1-3/Y1	E-Y1/E-Y	N/N	2-4	1.6-2	2.2-2.6	2.5
M6a	4	P/P	P/P	D/D	E-P/D-P	A-P/D-P	Y1-2/Y1-3	A-Y1/A-Y1	N/N	4-6	2.5-3	2.6-3.3	3
M6b	12	P/P	E-P/P	P/E-P	P/P	P-P	Y1-4/Y2-4	Y1/Y1-2	N/N	4-11	2.5-3	2.6-3.3	3.5
M7	4	P/P	P/P	A-P/A-P	A-P/A-P	A-P/A-P	Y2-3/Y2-4	Y1/Y2-3	N-A/E-A	6-11	3-3.33	3.2-3.4	3.8
M8A	6	P/P	P/P	P/P	P/P	P/P	Y2-3/Y2-3	Y2/Y1-2	Y1/Y1-2	8-13	>4	>3.2	4
M8B	5	P/P	P/P	P/P	P/P	P/P	Y3-4/Y3-4	Y1-3/Y1-3	Y1/Y1	9-15	>4	>3.2	4.5
M9	21	P/P	P/P	P/P	P/P	P/P	Y4/Y4-5	Y1-3/Y2-4	Y1-3/Y1-3	10-21	>4	>3.2	5
M10a	9	P/P	P/P	P/P	P/P	P/P	Y4-6/Y4-6	Y3-6/Y3-5	Y1-5/Y1-4	16-34	>4	>3.2	6
M10b	3	P/P	P/P	P/P	P/P	P/P	Y7/Y7-8	Y4-5/Y5	Y3-5/Y4	30-34	>4	>3.2	7.5
M11	2	P/P	P/P	P/P	P/P	P/P	Y9/Y8-9	Y6-8/Y5-8	Y4-5/Y4-5	36-38	>4	>3.2	9
M12	3	P/P	P/P	P/P	P/P	P/P	Y10/Y10	Y9/Y9-10	Y4-9/Y5-9	48-57	>4	>3.2	10
M14	1	P/P	P/P	P/P	P/P	P/P	Y12/Y11	Y11/Y11	Y11/Y11	67	>4	>3.2	12

Supplementary Table 5: **Estimated age classes of *C. sabaeus* Males** Monkeys were sorted into age classes based on the tooth eruption and degree to which dentine is exposed on molar occlusal surfaces. E Age refers to age based on dental eruption data and is given in months. E+W age is based on both dental eruption and molar wear and is given in years. Abbreviations given for teeth are: D=deciduous fully erupted, P=permanent fully erupted, E=gingival emergence, M=mesial half erupted, A=almost fully erupted, N=not erupted, and Y=molar present. Eruption and wear stages for upper teeth are given above and for lower teeth below the slanted line.

Age Class	N	I1	I2	C	P3	P4	M1	M2	M3	Sum Wear	E Age	E+W Age
F1a	2	D/D	D/D	D/D	D/A-D	E/E	N/N	N/N	N/N	0	0.4	0.4
F1b	2	D/D	D/D	D/D	D/D	D/D	N/N	N/N	N/N	0	0.4	0.4
F2	15	D/D	D/D	D/D	D/D	D/D	N-A1/A-Y1	N/N	N/N	2	0.2-1.2	1
F3a	2	D/D-P	D/D	D/D	D/D	D/D	Y1/Y1	N/N	N/N	2	0.2-1.2	1
F3b	5	P/P	D-P/E-P	D-E/D-M	D/D	D/D	Y1/Y1-2	N/N	N/N	2-3	2	1.5-2
F4a	4	P/P	P/P	D-A/A-P	D-A/A-P	D-P/D-P	Y1-3/Y2-4	A-Y1/A-Y1	N/N	3-7	2-3	2.5
F4b	7	P/P	P/P	D-P/E-P	A-P/A-P	P/P	Y2-4/Y3-4	A-Y1-2/Y1-3	N/N	3-11	2-3	2.5
F5	3	P/P	P/P	P/P	P/P	P/P	Y3/Y4	A-Y1/Y2-3	N/A-Y1	8-11	4	4
F6	3	P/P	P/P	A-P/P	A-P/A-P	P/P	Y3-5/Y5	Y1-2/Y3	E/R	10-14	4	5
F7	3	P/P	P/P	P/A-P	P/A-P	P/P	Y4-5/Y5-6	Y2-4/Y3-4	E-A/A-Y1	15-18	>4	7
F8	2	P/P	P/P	P/P	P/P	P/P	Y6-7/Y6-7	Y3-4/Y4	E-A/Y2	21-24	>4	12
F9	5	P/P	P/P	P/P	P/P	P/P	Y7-8	Y4-8/Y4-5	Y1-3/Y3-4	24-30	>5	13-15
F10	1	P/P	P/P	P/P	P/P	P/P	Y10/Y10	Y9/Y9	Y8/Y5	49	>5	17

Supplementary Table 6: **Estimated age classes of *E. patas* Females** Monkeys were sorted into age classes based on the tooth eruption and degree to which dentine is exposed on molar occlusal surfaces. E Age refers to age based on dental eruption data and is given in months. E+W age is based on both dental eruption and molar wear and is given in years. Abbreviations given for teeth are: D=deciduous fully erupted, P=permanent fully erupted, E=gingival emergence, M=mesial half erupted, A=almost fully erupted, N=not erupted, and Y=molar present. Eruption and wear stages for upper teeth are given above and for lower teeth below the slanted line.

Age Class	N	I1	I2	C	P3	P4	M1	M2	M3	Sum Wear	E Age	E+W Age
M1	6	D/D	D/D	D/D	D/D	D/D	N/N	N/N	N/N	0	0.4	0.4
M2a	1	D/D	D/D	D/D	D/D	D/D	N/A	N/N	N/N	0	0.4-1.4	1
M2b	9	D/D	D/D	D/D	D/D	D/D	Y1/Y1-2	N/N	N/N	1-3	0.4-1.4	1
M3	1	P/-	P/-	D/-	E/-	P/-	Y4/-	Y3/-	N/N	7(x2)	1-3	2.5
M4	1	P/P	P/P	P/P	P/P	P/P	Y4/Y-	Y2/Y-	Y1/Y-	7(x2)	4-5	4.5
M7	1	P/P	P/P	P/P	P/P	P/P	Y5/Y4	Y4/Y4	Y1/Y-	18+	>5	7
M9	2	P/P	P/P	P/P	P/P	P/P	Y3-6/Y6-7	Y3-6/Y3-5	Y1-5/Y1-4	28-31	>5	10-13
M10	1	M-P/P	M-P/P	P/P	P/P	P/P	Y11/Y-	Y8/Y-	Y5/Y-	24(x2)	>5	16

Supplementary Table 7: **Estimated age classes of *E. patas* Males** Monkeys were sorted into age classes based on the tooth eruption and degree to which dentine is exposed on molar occlusal surfaces. E Age refers to age based on dental eruption data and is given in months. E+W age is based on both dental eruption and molar wear and is given in years. Abbreviations given for teeth are: D=deciduous fully erupted, P=permanent fully erupted, E=gingival emergence, M=mesial half erupted, A=almost fully erupted, N=not erupted, and Y=molar present. Eruption and wear stages for upper teeth are given above and for lower teeth below the slanted line.

Covariate	Estimate (95% CI)
Intercept (β_0)	1263.49 (467.25, 3416.61)
Age	0.96 (0.94, 0.98)
Erythrocebus patas	0.99 (0.46, 2.13)
Papio papio	1.32 (0.80, 2.20)
Feb. Collection	1.16 (0.39, 3.43)
Mar. Collection	0.43 (0.18, 1.05)
Apr. Collection	0.55 (0.21, 1.45)
May Collection	0.61 (0.21, 1.79)
Dec. Collection	0.31 (0.08, 1.21)
Collected 2011	0.25 (0.13, 0.49)
Collected 2012	1.33 (0.71, 2.50)
RE Troop (b_0)	0.55 (-0.53, 1.64)
ICC	0.086

Supplementary Table 8: **Mixed Effects Regression on titer** Table reports the estimates from a mixed effects regression with CHIKV inverse $\log_2(\text{PRNT}_{80})$ as the outcome and monkey age, species, month of collection as fixed effects and troupe (same collection site and date) as a random effect. Intercept corresponds to a mean titer of 968 in the first year of life in *Chlorocebus sabaeus* monkeys collected in January, with the random effect indicating 95% of *Chlorocebus sabaeus* primate troupes collected in January have inverse PRNT_{80} titers between 124 and 7589 ($2^{(\beta_0 \pm 1.96 \cdot b_0)}$). ICC is the intraclass correlation for the random effect, and indicates about 8.6% of the total observed variance in titer is due to variance within monkey troops. Mixed effects models are preferred over fixed effects models by AIC (1652.8 versus 1802.2).

Supplementary Note 1: Details of Monkey Aging

Molar wear, the loss of enamel and exposure of underlying dentine on the occlusal surface due to contact with food and grit during chewing, was then used to place adult individuals with fully erupted permanent teeth into relative age categories. A standardized coding system for wear developed for fossil monkeys based on field research in baboons was utilized (Supplementary Figure 3 [1] and [2]). As monkeys age, occlusal wear changes from flattish wear facets without dentine exposed (wear stages 1-2), to dentine exposed progressively on one, two or more cusps wear (wear stages 3-8), to eventually no enamel being present on the occusal surface (wear stages 10-11). The sum of wear codes on all three molars was used to place adults into age classes. These discrete wear codes correspond well to percent dentine exposure (PDE) measures shown to increase steadily with advancing age in wild *Papio cynocephalus* and captive to wild Mandrillus sphinx whose birth dates and true ages were known as part of long term field research at Amboseli, Kenya and Lekedi Park, Gabon [3, 4]. Age of the oldest animals death in wild populations of baboons (28 years), African green monkeys (12 years), and patas (17 years) was assumed to be the age of adults with the most extreme tooth wear in this study [3, 5, 6]. Because male Papio papio in our study reached more advanced stages of molar wear than Papio cynocephalus, and males and female baboons of equavalent age in Amboseli had equivalent levels of PDE, we use the female age of death, 27 years, for our oldest male age rather than the 21 year oldest male age observed in Kenya [3]. Estimated chronological age for each molar wear class between age at M3 eruption and oldest age are based on dividing intermediate age classes into fairly equal time periods corresponding to progressive stages of wear. Supplementary Tables 3–7 give the number of primates in each age class, state of tooth eruption and wear and estimated ages. Note: not all monkeys had adequate dental casts or photos taken. These monkeys were aged based on anthropomorphic measurements (N=44 *C. sabaeus*, N=2 *E. patas*, and N=14 *P. papio*).

Supplementary Note 2: Inverse PRNT_{80} CHIKV titer

Associations with inverse titer are presented in Supplementary Table 8 and Supplementary Figure 3. Age was significantly negatively associated with inverse titer, with each year of age corresponding to about a 4% decrease in titer ($\beta = 0.96$ [95% CI, 0.94, 0.98]). Large differences in titer were seen across study years, with 2011 having 75% smaller titers ($\beta = 0.25$ [95% CI, 0.13, 0.49]). This is likely due to there being no inverse titers of 1280 observed in 2011.

Supplementary Note 3: Age-Varying Force of Infection

Supplementary Figure 5 shows estimates of $\lambda(t)$ by age of primate for years 2010, 2011, and 2012. Large spikes in $\lambda(t)$ are present for young monkeys. Interestingly, there are spikes in $\lambda(t)$ in about 8 year old monkeys.

Supplementary Note 4: Sensitivity of $\lambda(t)$ to Age-biased Monkey Collections

Estimates of $\lambda(t)$ from observed collections of monkeys may be biased due to being based on a sample that may not be representative of the true population. To explore the effects of differential sampling by age, we simulated primate populations and estimated $\lambda(t)$. Primate samples were simulated by drawing populations of 100 monkeys from an exponential distribution of ages (Supplementary Figure 8 top left panel) or a mixture distribution with a dip for juvenile ages (4-7 years old, Supplementary Figure 9, top left panel). We calculated the coverage probabilities of the bootstrap confidence intervals over a range of true $\lambda(t)$, and assessed the direction of bias. Supplementary Figures 8 and 9 summarize these sensitivity analyses. We find the coverage probabilities of confidence intervals to decrease as $\lambda(t)$ increases (Supplementary Figures 8 and 9, bottom left panel), though the coverage probabilities are better in the exponential case. We also find our method tends to over-estimate $\lambda(t)$ as the true $\lambda(t)$ increases in both cases (Supplementary Figures 8 and 9, bottom right panels).

References

[1] B. R. Benefit, "Phylogenetic, paleodemographic, and taphonomic implications of victoriapithecus deciduous teeth from maboko, kenva," *American journal of physical anthropology*, vol. 95, no. 3, pp. 277–331, 1994.

[2] J. E. Phillips-Conroy, *Dental variability in Ethiopian baboons: an examination of the anubis-hamadryas hybrid zone in the Awash National Park, Ethiopia.* PhD thesis, New York University, Graduate School., 1978.

[3] J. Galbany, J. Altmann, A. Pérez-Pérez, and S. C. Alberts, "Age and individual foraging behavior predict tooth wear in amboseli baboons," *American journal of physical anthropology*, vol. 144, no. 1, pp. 51–59, 2011.

[4] J. Galbany, A. Romero, M. Mayo-Alesón, F. Itsoma, B. Gamarra, A. Pérez-Pérez, E. Willaume, P. M. Kappeler, and M. J. Charpentier, "Age-related tooth wear differs between forest and savanna primates," *PloS one*, vol. 9, no. 4, p. e94938, 2014.

[5] N. Nakagawa, H. Ohsawa, and Y. Muroyama, "Life-history parameters of a wild group of west african patas monkeys (erythrocebus patas patas)," *Primates*, vol. 44, pp. 281–90, Jul 2003.

[6] L. A. Isbell, T. P. Young, K. E. Jaffe, A. A. Carlson, and R. L. Chancellor, "Demography and life histories of sympatric patas monkeys, erythrocebus patas, and vervets, cercopithecus aethiops, in laikipia, kenya," *Int J Primatol*, vol. 30, pp. 103–124, Feb 2009.
